# IVM Advances for Early Antral Follicle-Enclosed Oocytes Coupling Reproductive Tissue Engineering to Inductive Influences of Human Chorionic Gonadotropin and Ovarian Surface Epithelium Coculture

**DOI:** 10.3390/ijms24076626

**Published:** 2023-04-01

**Authors:** Alessia Peserico, Chiara Di Berardino, Giulia Capacchietti, Chiara Camerano Spelta Rapini, Liliana Liverani, Aldo Roberto Boccaccini, Valentina Russo, Annunziata Mauro, Barbara Barboni

**Affiliations:** 1 Department of Bioscience and Technology for Food, Agriculture and Environment, University of Teramo, 64100 Teramo, Italy; 2 Institute of Biomaterials, Department of Materials Science and Engineering, Friedrich-Alexander University of Erlangen-Nuremberg, 91054 Erlangen, Germany; 3 DGS S.p.A., 00142 Rome, Italy

**Keywords:** in vitro oocyte maturation, sheep, early antral follicle, follicle-enclosed oocyte, PCL electrospun scaffold, human chorionic gonadotropin, ovarian surface epithelium, EGF signaling

## Abstract

In vitro maturation (IVM) is not a routine assisted reproductive technology (ART) for oocytes collected from early antral (EA) follicles, a large source of potentially available gametes. Despite substantial improvements in IVM in the past decade, the outcomes remain low for EA-derived oocytes due to their reduced developmental competences. To optimize IVM for ovine EA-derived oocytes, a three-dimensional (3D) scaffold-mediated follicle-enclosed oocytes (FEO) system was compared with a validated cumulus-oocyte complex (COC) protocol. Gonadotropin stimulation (eCG and/or hCG) and/or somatic cell coculture (ovarian vs. extraovarian-cell source) were supplied to both systems. The maturation rate and parthenogenetic activation were significantly improved by combining hCG stimulation with ovarian surface epithelium (OSE) cells coculture exclusively on the FEO system. Based on the data, the paracrine factors released specifically from OSE enhanced the hCG-triggering of oocyte maturation mechanisms by acting through the mural compartment (positive effect on FEO and not on COC) by stimulating the EGFR signaling. Overall, the FEO system performed on a developed reproductive scaffold proved feasible and reliable in promoting a synergic cytoplasmatic and nuclear maturation, offering a novel cultural strategy to widen the availability of mature gametes for ART.

## 1. Introduction

In vitro maturation (IVM) of oocytes may represent the first step in the production of embryos in vitro, and it is essential for biomedical studies and assisted reproduction techniques (ART).

IVM is particularly attractive to selected specific infertile patients, such as those at risk of ovarian hyperstimulation syndrome (e.g., women with polycystic ovary syndrome, PCOS) and cancer sufferers who wish to preserve fertility prior to performing gonadotoxic treatment [1]. However, despite its advantages, clinical outcomes are still suboptimal, with IVM pregnancy rates significantly lower than IVF [2,3], probably ascribable to suboptimal or impaired cumulus cells metabolism [4], asynchronous oocyte nuclear and cytoplasmic maturation, and decreased developmental competence [5]. IVM is equally important if applied to veterinary medicine both for domestic animals by considering the value of the livestock industry and the urgency to safeguard threatened species [6]. Besides the veterinary IVM progress and application in the last years, the clinical outcomes of these IVM oocytes are also lower compared with those of oocytes matured in vivo [7].

To overcome this problem, different strategies have been adopted to improve IVM protocols to better support nuclear and cytoplasmic maturation occurring physiologically as a result of in vivo ovulatory stimuli [8,9]. Although several attempts have been made with oocytes derived from antral follicles, few studies have been performed with low competent ones with a proficient level of efficacy.

On the one hand, the strategies applied to oocytes derived from antral follicles include (1) setting up three-dimensional (3D) cultural systems [10,11,12,13,14], (2) testing the optimal duration of the in vitro culture [15,16], (3) IVM supplementation of growth factors (GFs) and molecules [17,18,19,20,21,22,23,24,25,26,27,28,29,30], (4) co-cultural supportive incubation with different reproductive-derived cells or stem cells [31,32,33,34,35,36,37,38,39,40,41,42,43], and (5) development of enclosed-follicle oocyte protocols [44,45,46,47,48,49,50].

On the other hand, the strategies tested to improve the performance of IVM of low competent oocytes were mainly focused on the use of dynamic cell culture devices (3D cultures) [51,52] and/or the supplementation of the IVM system with factors and/or molecules essential to the ovarian microenvironment [53,54]. Alternatively, the “simulated physiological oocyte maturation” (SPOM) protocol has also been applied to improve low competent oocyte IVM in bovine [55] and caprine [56,57,58] species. This technique is addressed to avoid spontaneous meiotic resumption to potentially synchronize nuclear and cytoplasmic maturation by overcoming the heterogenicity of the ovarian follicle population used [59] through a pre-maturation step aimed to block meiosis typically targets oocyte c AMP levels [60].

Results gathered from the above investigations hold great potential, as they might provide clear operational cues for developing an innovative system of in vitro maturation of immature oocytes.

In this context, the adoption of a more complex follicle-enclosed oocyte maturation (FEO) method might be promising if applied to early-stage follicles. FEO is an old IVM protocol adopted, firstly, to study the mechanism controlling meiotic quiescence and to reproduce the induction of meiosis (hamster [44], rat [45], rabbit [46], sheep [47,48], porcine [49]) physiologically. A strategy that initiates with the hormonal triggers and then propagates throughout the somatic compartment of the follicle by the EGF-like peptides (amphiregulin, epiregulin, and betacellulin) has recently been re-proposed to better mimic and synchronize the LH-inducing of oocyte maturation mechanisms [61]. This technological strategy has been mainly applied to low-competent mice oocytes derived from in vitro follicle development (IVFD) [62], whereas it has been less adopted in large mammal models.

Improving the efficiency of IVM becomes still more relevant when the reproductive technology is applied to low-competent oocytes derived from early antral follicles, particularly those derived from IVFD or surviving after the cryopreservation of ovarian tissue [62]. The possibility of recapitulating the completion of meiosis and the developmental competence by maintaining genetic integrity in oocytes derived from early-stage follicles would surprisingly broaden the scope of application of IVM by answering the unresolved demand to succeed with ART in preserving women’s fertility [63,64,65] to amplify the heritage of genetically important livestock breeds [66,67] and to propagate endangered species [68].

Currently, only MII oocytes derived from in vitro-developed rodent follicles have displayed adequate developmental competence by producing live offspring, even if in a very limited number [69,70,71], while those derived from in vitro-developed domestic animal or non-human primates have so far proved to be exclusively able to generate preimplantation embryos [62].

With the final aim to broaden ART, another relevant biological variable to face is the age of the donor.

However, the IVFD technique represents a potential strategy to preserve female fertility, considering that the biomedical interest in this advanced reproductive technique has been further increased recently, consequently to the higher chance of survival for oncological patients diagnosed in pre-pubertal age [72]. Instead of transplanting patients’ ovarian tissues, which could expose the organism to the risk of reintroducing cancerous cells, setting up validated IVFD protocols could represent a safe biotechnical strategy for restoring fertility chances once patients reach adulthood in a healthy condition [73,74].

Juvenile in vitro embryo transfer (JIVET) is also a promising technology with the greatest advantage of rapidly expanding excellent livestock breeds and reducing the generation intervals in breeding programs [75]. However, the oocytes from juvenile animals exhibit much lower developmental ability than that from adult animals [76], and the possibility of improving their developmental competence remains to be further explored.

In order to place IVM in the spectrum of fertility preservation strategies for low-competent oocytes derived from juvenile lamb early-stage follicles, the present research has been designed to assess the possibility of exploiting modern-engineered reproductive tissues and culture systems to improve IVM outcomes.

To this aim, IVM of a follicle-enclosed oocyte (FEO) derived from early antral follicles (EA) was performed on patterned poly(epsilon-caprolactone) (PCL) electrospun scaffold, a fibrous construct composed of fibers mimicking the natural morphology of the ovarian tissue, obtained by following a bioinspired approach [77,78]. The feasibility of a biomimetic 3D IVM system was tested by assessing the oocyte in vitro meiotic competence and parthenogenetic activation (MII and parthenogenetic activation rates) by comparing the reproductive FEO outcomes with those obtained under a validated IVM system specifically developed for lamb low competent oocytes [39,78,79,80,81]. The comparative IVM approach has been further exploited to test the role of gonadotropin stimulation (eCG + hCG) and/or cell-based cocultures to generate a controlled IVM system standardized for low-competent oocytes.

## 2. Results

### 2.1. IVM of EA-Derived Oocytes: Role of Chorionic Gonadotropin and/or Cells Cocultures

#### 2.1.1. Role of Chorionic Gonadotropin

**Figure 1:**
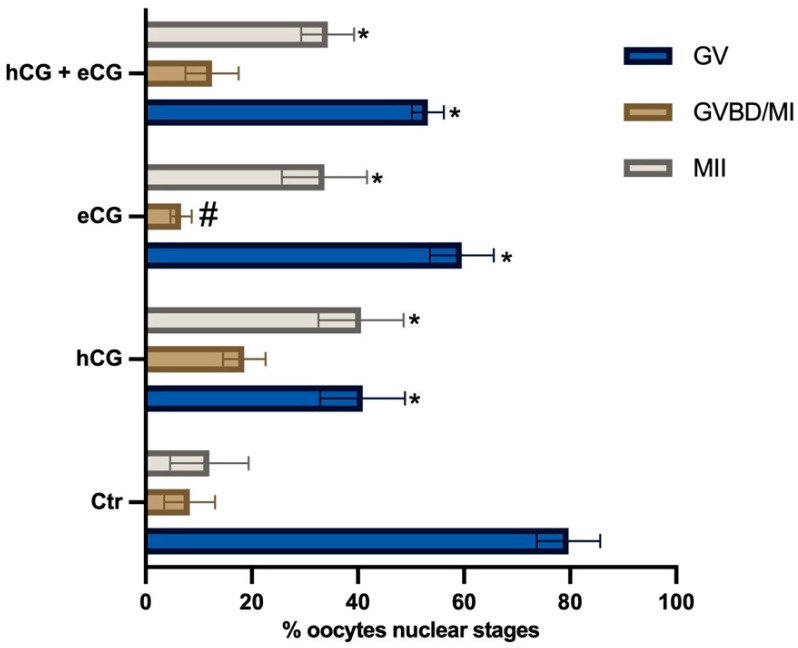
Percentage of meiotic competent IVM EA-derived oocytes: role of chorionic gonadotropin stimulation. Three independent biological replicates were performed for each experimental group (total number of oocytes analyzed: 132, 134, 128, and 130, respectively, for Ctr (without CG), hCG, eCG, and hCG + eCG). Both CGs were used at 5 IU/mL. The significant data (from *p* < 0.05) analyzed vs. Ctr (without CG) or vs. 5 IU/mL hCG were indicated with * and ^#^ superscripts, respectively.

To determine the role of chorionic gonadotropin (CG) stimulation in triggering meiotic resumption in IVM of low competent EA-derived oocytes, cumulus-oocyte complex cultures were performed in the presence or absence of eCG and/or hCG (5 IU/mL for both). The results summarized in Figure 1 demonstrated that hormonal stimulation was essential to trigger oocyte maturation. Low-competent oocytes resume meiosis spontaneously once removed from the EA follicles in a very low percentage (GV = 80% without CG vs. 41% hCG vs. 59.6% eCG vs. 53.2% hCG + eCG: for all *p* < 0.05). Moreover, using CG alone or in combination promoted a similar rate of metaphase II stage oocytes (MII: 40.6% hCG vs. 33.7% eCG vs. 34.3% hCG + eCG: *p* > 0.05 vs. 34.3% in hCG + eCG, respectively).

However, the hormonal treatments displayed a different ability to promote meiosis. Indeed, hCG demonstrated an overall higher efficiency in triggering the resumption of meiosis in EA-derived oocytes (GVBD/MI + MII oocytes: 59.2% hCG vs. 40.4% eCG vs. 46.8% hCG + eCG: for *p* < 0.05, respectively).

Based on the results above, 5 IU/mL hCG was used in the following experiment to test the influence of cell coculture.

#### 2.1.2. Role of Somatic Cell Cocultures

**Figure 2:**
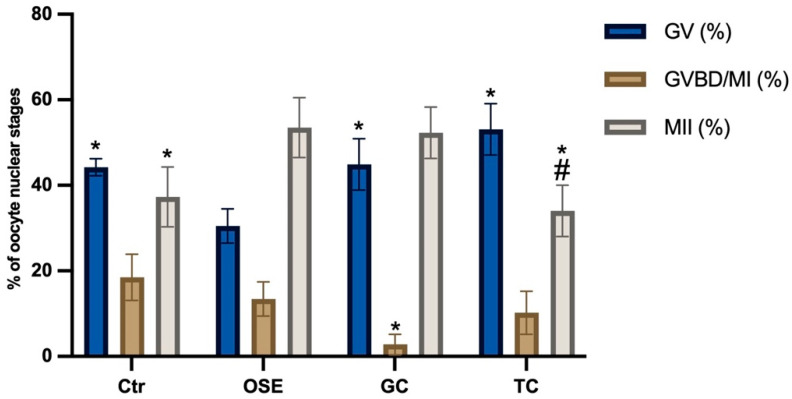
Influence of somatic cell cocultures during IVM of EA-derived oocytes. The IVMs were performed with hCG in the absence (Ctr) or presence of primary cell monolayers. The used ovine cell typologies were ovarian epithelial cells (OSE), granulosa cells (GC), or tenocytes (TC). Three independent biological replicates were performed for each experimental group (total number of oocytes analyzed: 129, 127, 129, and 126, respectively, for Ctr, OSE, GC, and TC. The significance of nuclear stage data (*p* < 0.05) was analyzed vs. Ctr (in the absence of somatic cells coculture) or vs. OSE and indicated with (*) or (#) superscripts, respectively.

To evaluate the influence of somatic cells on the maturation of EA-derived oocytes, IVM was triggered with hCG (5 IU/mL) and carried out in the absence (negative Ctr) or presence of primary cell cocultures. To this aim, three different cell typologies were compared: two derived from the ovary (OSE and GC) and one from a non-reproductive system (TC). Exclusively, the IVMs performed in coculture with ovarian-derived cells, independently of typology (OSE and GC), were able to significantly increase the percentage of MII oocytes (MII: 37.3% in Ctr vs. 53.5% with OSE vs. 52.3% with GCs: for both *p* < 0.05) Figure 2). On the contrary, TCs coculture did not exert any additional inductive role over Ctr (MII: 37.3% in Ctr vs. 34% with TC: *p* > 0.05).

Although the rate of MII oocytes was comparable between OSE and GC (see Figure 2), OSE cocultures promoted a significantly greater overall resumption of meiosis (GVBD/MI + MII oocytes: 67% vs. 55%; *p <* 0.05).

### 2.2. EA Follicles Enclosed Oocytes (FEO) Maturation: Role of hCG and/or Somatic Cells Cocultures

#### 2.2.1. FEO Maturation: Set Up of CGs Stimulation

The advanced cultural systems based on an engineered reproductive approach were adopted to perform IVM using ovine FEO.

**Figure 3:**
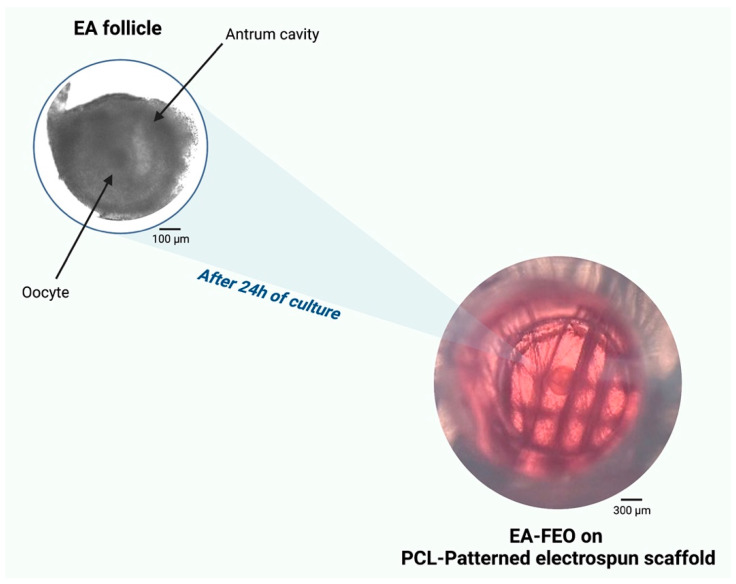
An example of EA incubated as FEO for 24 h on PCL-Patterned electrospun scaffolds in the absence of any hormonal stimulation.

**Figure 4:**
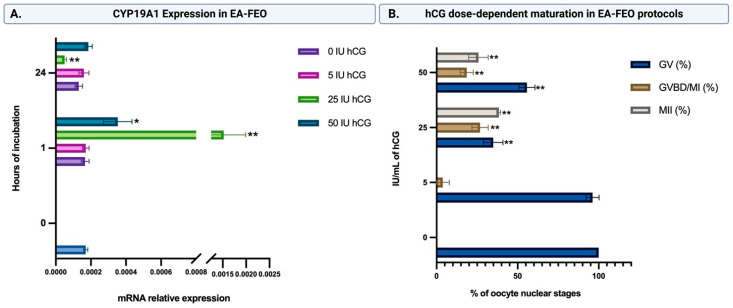
(**A**) Influence of hCG stimulation of FEO on the expression of steroidogenic-specific gene CYP19A1. The maturation protocols were optimized for hCG stimulation by carrying out the EA-FEO incubation on PCL scaffolds for 24 h and exposing them to increasing doses of hCG (5, 25, 50 IU/mL) or without any hormonal stimulation (Ctr). The responsiveness of FEO to hCG was functionally tested by comparing the CYP19A1 gene expression before (time 0, Ctr) and after hCG stimulation (at 1 and 24 h). Three independent biological replicates were performed. Ten follicular walls per group were processed for gene expression analysis. mRNA data were statistically analyzed vs. Ctr (Time 0) and indicated with (*) or (**) when significantly different for *p* < 0.05 or *p* < 0.01, respectively. (**B**) hCG influence of FEO meiotic resumption. The induction of maturation promoted by different doses of hCG (0, 5, 25, 50 IU/mL) was tested in EA-FEO after 24 h of incubation by analyzing the oocyte nuclear stages. Three independent biological replicates were performed for each experimental group (total number of oocytes analyzed: 126, 132, 127, and 133, respectively, for time 0, 5, 25, and 50 IU/mL). The significance of each nuclear stage data (GV, GVBD/MI, or MII) was statistically analyzed vs. Ctr (0 IU hCG) and indicated with (*) or (**) superscripts when *p* < 0.05 or *p* < 0.01, respectively.

Firstly, FEO incubation on PCL scaffolds was tested for 24 h by demonstrating that this incubation was compatible with the preservation of three-dimensional organization on a valuable experimental sample (three replicates of 24 EA follicles each). The quality of FEO cultured structures was confirmed by the healthy morphological aspect of both the somatic and germinal compartment (Figure 3): the presence of an antral cavity, compactness of mural and cumulus cells, absence of cytoplasm darkness in both somatic and germinal cells. From a functional point of view, the oocytes derived from FEO and cultured on PCL scaffolds in the absence of any CG stimulation maintained the meiotic arrest by displaying the GV nuclear stage in the totality of cases (Figure 4B). The COCs isolated from cultured FEO remained morphologically intact in more than 85% of the cases.

In order to trigger intrafollicular maturation, hCG stimulation was set up. To this aim, a dose-effect curve was performed starting from the validated dose of hCG used for the IVM system (5 IU/mL). The influence of hCG on FEO was tested by analyzing the modulation of CYP19A1 expression at 1 h and 24 h and by recording the MII rate at the end of incubation.

A time-dependent regulation of CYP19A1 expression was substantially obtained exclusively in FEO exposed to 25 IU/mL hCG, where an early activation of the steroidogenetic gene followed by a drastic final inhibition was recorded.

More in detail, FEO exposed to 25 IU/mL hCG for 1 h displayed a peak in CYP19A1 expression that was 8.9-fold higher than the pre-treatment transcripts values (25 IU/mL hCG vs. Ctr; *p* < 0.01). Then, CYP19A1 mRNA displayed a marked decrease reaching a value at 24 h 3.4-fold lower than Ctr (*p* < 0.05: Figure 4A). The levels of CYP19A1 mRNA did not change when FEOs were exposed to the other hCG doses, with the only exception of FEO treated with 50 IU/mL hCG for 1 h (Figure 4A).

At the same time, 25 IU/mL hCG was also able to promote the highest rate of MII oocytes (Figure 4B).

Notably, almost all oocytes incubated for 24 h in FEO systems maintained the condition of meiotic arrest up to a dose of 5 IU hCG/mL (Figure 4B). Instead, the induction of meiosis was recorded in 25 and 50 IU hCG-treated FEO. However, the highest percentage of MII oocytes was obtained with 25 IU/mL hCG (MII: 38.4% in 25 IU vs. 25.8% in 50 IU/mL hCG; *p <* 0.01). Consistently, the 50 IU/mL hCG-treated group also showed a higher percentage of GV oocytes (GV: 55.7% vs. 34.9% in 25 IU/mL hCG; *p <* 0.01).

**Figure 5:**
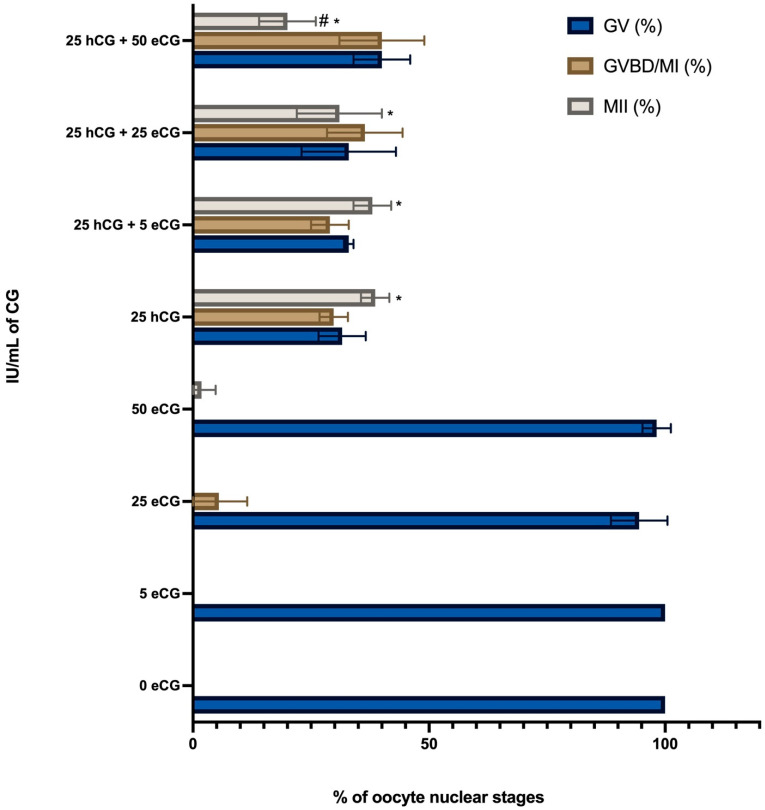
Influence of eCG stimulation of FEO on oocyte meiotic resumption. The experiments were performed by exposing EA-FEO to increasing doses of eCG (0, 5, 25, 50 IU/mL) in the absence or presence of hCG (25 IU/mL). Three independent biological replicates were performed for each experimental group (total number of oocytes analyzed: 95, 95, 100, and 94 respectively for 0, 5, 25, and 50 IU/mL eCG and 99, 101, 101, and 94, respectively, for 25 IU/mL hCG, 25 hCG +5 eCG, 25 hCG +25 eCG, 25 hCG + 50 eCG IU/mL). The significance of each nuclear stage data (GV, GVBD/MI, or MII) collected in dose-response eCG stimulation was statistically analyzed vs. 0 eCG indicated with (*) superscript when *p* < 0.05. The significance of each nuclear stage data (GV, GVBD/MI, or MII) collected in dose-response eCG stimulation in the presence of 25 IU/mL hCG was statistically analyzed vs. 0 eCG + 25 hCG indicated with (#) superscript when *p* < 0.05.

Then, the contribution of eCG in the FEO cultural system was also tested alone or in combination with 25 IU/mL of hCG (Figure 5).

The gathered data confirmed that eCG did not induce maturation up to 50 IU/mL as well as it did not exert any synergic action when combined with hCG.

The results showed that 25 IU/mL hCG was used in the following experiments designed to evaluate the effect of cell cocultures in IVM performed using FEO.

#### 2.2.2. FEO Maturation: Role of Cells Cocultures on Oocyte Maturation

**Figure 6:**
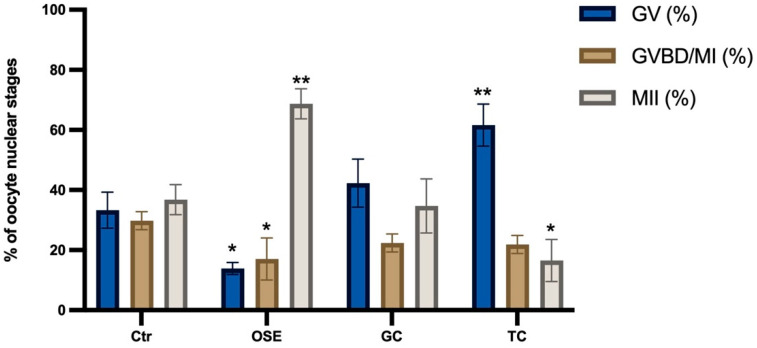
Influence of somatic cell cocultures on EA-FEO maturation protocol. The EA-FEO were induced to mature with hCG (25 IU/mL) in the absence (Ctr) or presence of primary cell monolayers. The used ovine cell typologies were: ovarian surface epithelial cells (OSE), granulosa cells (GC), or tenocytes (TC). Three independent biological replicates were performed for each tested experimental group (total number of oocytes analyzed: 131, 126, 129, and 128, respectively, for Ctr, OSE, GC, and TC). The significance of nuclear stage data (*p* < 0.05) was analyzed vs. Ctr (hCG without cells) and indicated with (*) or (**) for *p* < 0.05 and *p* < 0.01 superscripts, respectively.

To investigate whether ovarian-derived (OSE and GC) or non-ovarian-derived (TC) cells could influence FEO cultural performances as previously demonstrated for IVM protocol, the rate of oocyte maturation was evaluated at the end of culture carried out with the hCG stimulatory influence (25 IU/mL hCG) and in the absence (Ctr) or presence of primary cell monolayer (Figure 6).

Interestingly, the assessment of the oocyte nuclear stage revealed that FEO cocultured with OSE significantly improved the resumption of meiosis, almost doubling the MII rate over the Ctr condition (MII: 68.7% OSE vs. 36.8% Ctr; *p <* 0.01) and over both GC and TC co-cultural groups (34.7% and 16.5%: for both *p <* 0.01 vs. OSE coculture group). Of note, TC exerted a clear adverse effect by inhibiting the triggering hCG influence (GV: 33.3% Ctr vs. 61.6% TC; *p* < 0.01)

#### 2.2.3. FEO Maturation: Influence of Cell Coculture on Steroidogenesis and on hCG-Inducing Maturation Pathways

To assess the role of somatic cell coculture in mediating the inductive maturation influence of hCG, the effect of CG on the expression of CYP19A1 and EGFR signaling pathway activation was analyzed.

**Figure 7:**
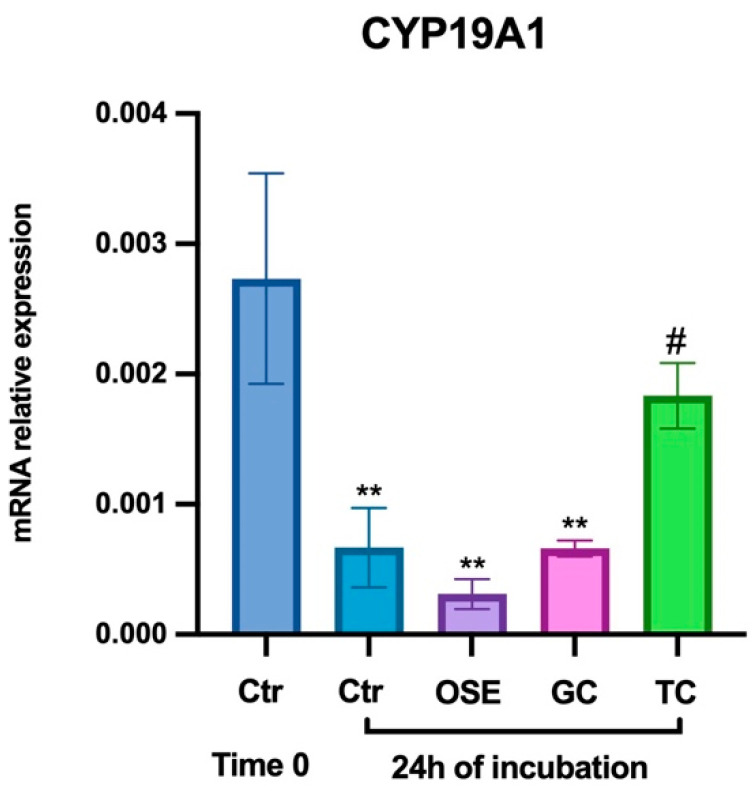
Role of somatic cell coculture on CYP19A1 expression. Three independent FEO maturation protocols were analyzed in the absence (Ctr: hCG alone) or in the presence of primary somatic cell cocultures. Ten follicular walls per group were processed for gene expression analysis. The significance of mRNA values (*p* < 0.05) was statistically analyzed vs. Ctr Time 0 or vs. Ctr 24 h of incubation and indicated with (**) or (#) superscripts for *p* < 0.05, respectively.

Concerning the gene expression data, steroidogenic transcriptional program shutdown was recorded in all tested maturation conditions with different levels of efficiency (Figure 7).

In the absence of any cocultures (Ctrl 24 h), hCG alone induced a fourfold downregulation change of CYP19A1 (vs. Ctr time 0; *p <* 0.01). A significant downregulation trend was recorded in both FEO cocultured with GC and OSE (4-fold and 8.7-fold, respectively: both vs. Ctr time 0, *p <* 0.01). On the contrary, any notable reduction was detected in FEO cocultured with TC (1.5-fold change decrease vs. Ctr time 0: *p* > 0.05). In addition, in the presence of TC, the levels of CYP19A1 transcripts at the end of incubation were significantly higher than those recorded in the other cultural conditions (vs. Ctr vs. OSE vs. GC: for all *p* < 0.05).

**Figure 8:**
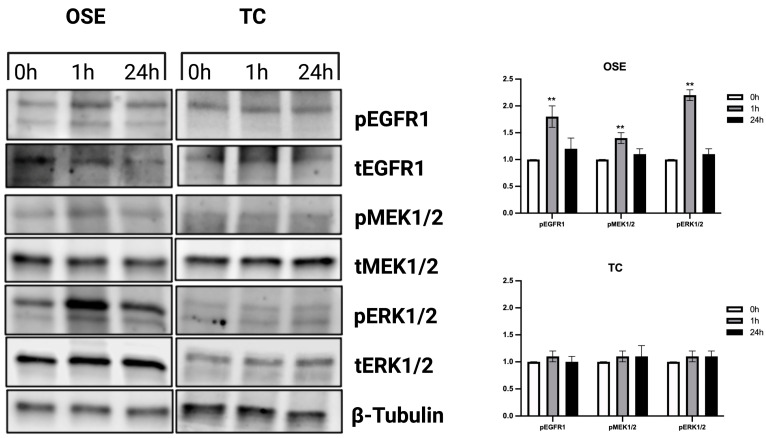
Time course of EGF signaling cascade in FEO cultured in the presence or absence of OSE and TC. Immuno-blot analysis of EGF signaling cascade phospho-activation in FEO with OSE and TC was performed at 0, 1, and 24 h. Three independent biological replicates were performed, and a representative blot is shown. Eight follicular walls per group were processed and collected at 0, 1, and 24 h of FEO. Protein quantification graphs report media of signals detected in the three tested biological replicates. The significance of protein expression values (*p* < 0.05) was statistically analyzed vs. 0 h of FEO and indicated with (**) for *p <* 0.01.

EGF signaling pathway activation was then assessed in FEO with the poorest performances in terms of steroidogenic inhibition (FEO with TC) and FEO with OSE, selected as representative of the ovarian-derived cells type category (Figure 8). When FEO was carried out in the presence of OSE, a marked and significant activation of EGF cascade was observed at an early time of incubation (1 h), followed by a sharp decrease up to the basal level at 24 h (see Figure 8). Indeed, at 1 h, EGFR and its downstream intracellular MAPK mediators, such as MEK1/2 and ERK1/2, showed respectively 1.8-fold; *p <* 0.01, 1,4-fold; *p <* 0.01 and 2.2-fold; *p <* 0.01. The time-dependent activation of the EGF pathway was not detected when FEO was performed with TC in coculture.

#### 2.2.4. Comparison of FEO vs. IVM Protocols in Coculture

A comparative study was finally performed to assess the efficacy of IVM or FEO maturation systems carried out with the validated gonadotropin stimulation (5 and 25 IU/mL for IVM and FEO, respectively) and the cell coculture protocols.

**Figure 9:**
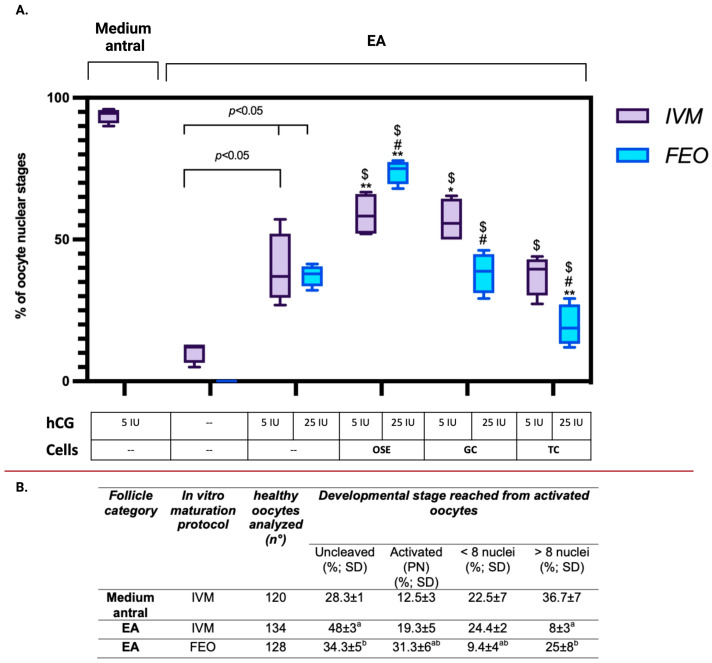
(**A**) Comparison between somatic cells’ coculture effect on FEO and IVM protocol. Four independent biological replicates were performed for each tested experimental group (total number of oocytes analyzed: 136 and 134 respectively for IVM and FEO Ctr without CG, 148 and 138 for IVM and FEO Ctr without cells coculture, and 143 and 144 for IVM and FEO with OSE, 140 and 142 for IVM and FEO with GC, and 135 and 137 for IVM and FEO with TC). The significance of MII data collected in different cell-coculture experimental groups was analyzed within each protocol (IVM or FEO) vs. EA-derived oocytes as negative Ctr (5 IU/mL hCG or 25 IU/mL hCG without cells, respectively, for IVM and FEO) and indicated with (*) or (**) superscript for *p* < 0.05 or *p* < 0.01. The significance of MII data collected in different cell-coculture experimental groups was analyzed within each protocol (IVM or FEO) vs. medium antral-derived oocytes as positive Ctr (5 IU/mL hCG without cells for IVM) and indicated with ($) superscript for *p* < 0.05. The significance of MII data between the two protocols (IVM vs. FEO) in each cell-coculture experimental group (OSE or GC or TC) was analyzed and indicated with (#) superscript for *p* < 0.05. (**B**) The developmental stage reached from parthenogenetic oocytes activation after 72 h of incubation. The parthenogenetic activation was performed on MII oocytes (extruded PB) obtained from IVM, and FEO optimized EA protocols (with OSE and hCG) and compared with that of IVM oocytes derived from medium antral follicles (positive Ctr). Three independent biological replicates were performed for each tested experimental group (total number of oocytes analyzed: 120, 134, and 128, respectively, derived from medium antral, IVM, and FEO protocols). The data were statistically analyzed, and the significant values for *p <* 0.05 were indicated with ^a^ vs. medium antral; ^b^ vs. EA IVM.

As summarized in Figure 9A, the FEO protocols resulted in a more controlled system where the oocytes maintained a complete meiosis arrest in the absence of any hormonal stimulation (any spontaneous resumption of meiosis). Additionally, even if the induction of maturation promoted by hCG was only slightly higher than that obtained with IVM (MII: 39.5% Ctr IVM vs. 37.3% Ctr FEO; *p* > 0.05), however, the maturation outcomes were more repeatable (SD: ±13 vs. ±4 for Ctr IVM and Ctr FEO, respectively).

Moreover, the stimulatory influence of somatic cell coculture on maturation resulted in being either cell- or protocol-dependent.

Only ovarian-derived cells were able to exert an inductive maturation effect in both IVM and FEO. However, amongst the reproductive-derived cells, the GCs were able to positively influence the induction of maturation exclusively in IVM (see Figure 9A), whereas OSE exerted an inductive influence in both the maturation protocols.

Indeed, the resumption of meiosis was significantly improved in IVM when the low competent oocytes were incubated alternatively with GC or OSE instead of without cells (MII: 58.8% in OSE and 53.8% in GCs vs. 39.5% Ctr; *p <* 0.05). Differently, only OSE coculture was able to improve the rate of MII oocytes over the Ctr conditions (without cells and with hCG) in FEO protocols (MII: 74% with OSE vs. 37.3% in Ctr *p* < 0.05), whereas GC did not exert any effect (MII: 38.3% with GC vs. 37.3% Ctr: *p* > 0.05).

Of note, OSE efficiency in inducing MII oocytes was significantly greater in the FEO maturation system (MII: OSE IVM vs. OSE FEO: *p* < 0.05).

Moreover, TC did not add any ameliorative effect in both protocols. Indeed, the percentage of MII oocytes never exceeded the value recorded under the Ctr condition (without cells and with hCG) in TC-IVM (MII: 37, 6% with TC vs. 39.5% Ctr; *p* > 0.05). On the contrary, TC exerted a negative impact on MII oocytes in the FEO system (MII: 19.7% with TC vs. 37.3% Ctr; *p* < 0.05 (see Figure 9A).

Finally, the developmental competence of the EA-derived oocytes matured by adopting the optimized FEO (FEO-OSE co-cultural system with 25 IU/mL hCG) and IVM protocols (IVM-OSE with 5 IU/mL hCG: Figure 9A) were tested. 

In order to assess the exclusive quality of oocyte cytoplasm, the activation was induced by using a chemical stimulus (ethanol) and not through fertilization. The parthenogenetic activation was assessed after 3 days and compared with those of fully grown oocytes derived from medium-antral follicles used as positive Ctr (Figure 9B).

FEO matured oocytes showed an activation rate significantly higher (65.7%) than that of IVM ones (51.7% *p <* 0.01) and only slightly lower than of IVM medium antral-derived oocytes (71.7%: *p >* 0.05)

A positive influence of the FEO-OSE maturation system on the parthenogenetic competence was also confirmed by the analysis of their more advanced activation stage. Approximately 25% of 128 oocytes derived from FEO-OSE maturation protocols displayed more than eight nuclei instead of 8% of 134 IVM ones (*p* < 0.01). Notably, a significantly high number of FEO-OSE matured oocytes were activated but never overcame the PN stage (Figure 9B).

However, the analysis of the activation stage clearly demonstrated the lower developmental competence of FEO-OSE-derived parthenotes compared to the Ctr ones. Approximately 60% of Ctr parthenotes are in an advanced stage of cleavage (< or > eight nuclei), whereas approximately 34% of FEO-OSE-derived oocytes reached this stage of activation (*p <* 0.05).

## 3. Discussion

This work demonstrated that EA could offer a source of ovarian follicles providing an additional number of oocytes to be potentially enrolled in IVM/IVF/EC protocols.

The low competent female gametes enclosed in EA follicles represent, indeed, a consistent amount of oocyte reserve, which can be derived directly from the ovary of several domestic animals collected to the slaughterhouse or, alternatively, derived by taking advantage of the progress in reproductive biotechnologies. Indeed, they can either survive after cryopreservation procedures [62] or obtain from the advanced protocol of in vitro folliculogenesis (*iv*F) [63,64,65]. The possibility to draw on the gamete reserve present in follicles at an early stage of development reinforces the ART success in preserving human fertility [82,83,84] and, for what concerns the veterinary medicine field, in amplifying the heritage of genetically important livestock breeds [66,67] or in contrasting the threat of extinction of several endangered mammalian species [68].

As demonstrated in the present study, in order to enroll the oocytes derived from EA for ART purposes, it is relevant to adapt the IVM protocols available to date for these low-competent gametes by combining the improved knowledge on oogenesis with the advances in cell culture procedures.

To fulfill this aim, the present research has taken advantage of the use of electrospun fibers composed of biodegradable biomaterials developed for reproductive tissue engineering (REPROTEN) applied to the ovarian tissue [78,85,86] or, alternatively, for performing 3D *iv*F (in vitro folliculogenesis) protocols aimed to grown PA follicles in culture up to the EA stage [78].

Based on the present results, first, it appeared evident that gametes derived from EA follicles required a specific gonadotropin control in order to be activated, as demonstrated in both FEO and IVM systems.

Consistently, the present data showed how the elective use of hCG, an LH-like chorionic gonadotropin, is the unique hormonal stimulus enabling the resumption of meiosis, whereas eCG, a more FSH-like chorionic gonadotropin, did not exert any action either alone at high dose or in combination with hCG despite the contradictory literature evidence (ruminants: [87,88,89,90,91]; porcine: [92,93]; human: [94,95,96,97,98]; mouse: [44,99,100,101,102,103,104,105,106].

Consistently, in this study, the induction of maturation in EA follicles requires specifically LH/hCG either in the FEO or in those released from EA follicles. Indeed, the maturation did not occur spontaneously in these low competent female gametes except in a very low percentage of oocytes exclusively under IVM protocols.

The maturation signal, in addition, was substantially enhanced in the presence of specific primary somatic cells, demonstrating that the paracrine signals released into the cultural environment are essential in supporting the activation of follicles and, indirectly, of germinal resumption of meiosis.

Consistently with literature IVM evidence collected to date, somatic/stem cell cocultures may be exploited for supplying paracrine factors enabling the oocytes to potentiate the maturation performances. The direct or indirect (conditioned media) presence of somatic/stem cell sources has been associated with the release of a variety of growth factors and cytokines, including Vascular-Endothelial Growth Factor (VEGF), basic Fibroblast Growth Factor (bFGF), Insulin-like growth factor-1 (IGF-1), Interleukin 10 (IL-10), and EGF that may overall impact on the signaling cascade events triggering the phase of maturation [35,107,108,109].

However, for the first time, this study demonstrated that this effect is strictly cell-specific.

The ability of granulosa cells derived from medium antral follicles to compensate for the immaturity of COCs derived from EA is not surprising [110,111]. This had already been demonstrated in previous work [39], where the lack of synchronicity between germinal and follicle cell development was identified as responsible for the maturation failure in the oocytes derived from ovine small antral follicles. This defect had been overcome by adding in coculture granulosa cells deriving from medium antral follicles that were able to supply for such immaturity by externally driving the intracellular signaling cascade of the events leading to meiosis resumption.

Conversely, for the first time, the great specificity of OSE in supporting FEO maturation is documented. This result has not only made it possible to validate a new protocol to foster the expression of maturation competence in oocytes derived from EA but, at the same time, this cultural system demonstrated the existence of two levels of intrinsic EA immaturity responsible for the oocyte maturation failure induced by LH/hCG, one involving the cumulus cells and the other the mural compartment. Indeed, even if it is known that OSE cells are capable of being affected by the LH stimulus, it has not yet been demonstrated that their exposure to LH-like hormone (hCG) activates mechanisms guiding the resumption of meiotic maturation into oocytes by acting exclusively through the stimulation of the mural follicle layer operative into FEO system.

As confirmed by earlier investigations, OSE cells express the LHCGR receptor [112,113,114]. They are able to reply to the LH/hCG stimulus by upregulating the expression of growth factors and/or their receptors which have been recognized as intraovarian regulators of oocyte maturation, such as members of the Insulin-like growth factor (IGF) and epidermal growth factor (EGF) family.

An increased level of IGF-1 transcript was identified upon LH/hCG stimulus in human [113] and ovine [115] OSE cells. Upregulation of the EGF receptor (EGFR) was observed upon LH stimulus in human immortalized OSE cells [116,117]. In both cases, gonadotropin stimulus was associated with an increased rate of OSE cell proliferation.

In the present in vitro model, OSE cells exclusively operate in the FEO system by activating the main intracellular signaling involved in the oocyte maturation [26]. Indeed, hCG induced in follicle walls of FEO a transient activation of the EGFR together with its downstream intracellular mediators, such as MEK1/2 and ERK1/2 kinases, exclusively when OSE are present, while this pathway was not activated in coculture with other sources of cells like tenocytes.

However, the present study did not investigate the upstream factors involved that have been identified in EGF family members, including amphiregulin, epiregulin, and beta-cellulin [61,118]. It must be considered that OSE might have a direct effect on the germinal compartment. However, the signals released by the OSE manage a clear dialogue with the somatic compartment’s mural layer, leading to the enhancement of the maturation performances when OSE are co-cultivated in the presence of FEO instead of COC.

Overall, the present data clearly indicate that EGF signaling is activated at a higher level in OSE when compared with TC. Based on these premises, a mechanistic approach aimed at the investigation of the crosstalk dynamics governing the paracrine signaling supported by OSE cocultured with FEO assumes high priority and deserves further research efforts as it might allow for the hypothesizing of target interventions to be applied with the aim of ameliorating the maturation performance of the immature ovarian follicular source to be enrolled for ART purposes.

The FEO system can be run with a high level of efficiency by exploiting the materials developed for REPROTEN. These innovative bioengineering approaches potentially enable the increasingly physiological in vitro modeling of reproductive key events availing of the use of a biocompatible scaffold to encapsulate the isolated follicles and the ovarian-derived cells, whose co-presence may be relevant for the survival and the maintenance of synchronous crosstalk between germinal and somatic follicular compartments. By exploiting the use of the PCL electrospun fibers applied for the fabrication of engineered reproductive materials [77,78,85,86], follicle culture systems can be developed by preserving the unaltered 3D structure, avoiding oocyte manipulation. This operative step can easily mechanically compromise oocyte integrity [119].

Accordingly, this study, employing the above PCL-scaffold, made it possible to propose a new in vitro maturation system for low competence oocytes and allowed to maximize the IVM efficiency in terms of MII rate and parthenogenetic oocytes activation by combining adequate gonadotropin stimulation with hCG in coculture with primary OSE cells. Of note, when FEO was cocultured in the presence of OSE cells, the resumption of meiosis greatly improved over the Ctr condition, nearly doubling the MII rate and over both GC and TC co-cultural groups.

Finally, it merits emphasis that this advanced FEO protocol could activate meiosis resumption in most oocytes and produce high-quality oocytes, as confirmed by the parthenogenetic activation performance. However, even if more than 60% of OSE-FEO were activated, the parthenogenetic activation rate remained lower medium-antral follicle-derived oocytes (71.7%: *p >* 0.05; see Figure 9B), in particular, considering the percentage of them reaching the advanced stage of cleavage.

Indeed, most of the zygotes were blocked to the PN stage in OSE-FEO-derived parthenotes. This might be indicative of reduced cytoplasmic quality, which results in cytoplasmic changes that take place during oocyte maturation, affecting organelle function and cytoskeletal integrity, as well as the machinery regulating intracellular activation mechanisms (i.e., Ca^2+^ storage or intracellular Ca store responsiveness). Notably, the pronuclei are formed in a process that is also dependent on Ca^2+^ levels and involves the MAPK pathway [120,121]. Thus, deficiencies in this transduction pathway could prevent the oocyte from undergoing proper embryogenesis [121]. However, techniques aimed at improving pronuclear manipulation and quality, as well as at optimizing the culture environment to help maintain embryo developmental potential, are still under investigation [122]. Some indications for the amelioration of embryo development might be obtained from oocyte pronuclear manipulation in mouse models where preliminary data postulate that abnormal pronuclear development could determine some detrimental epigenetic effects [123,124].

These aspects merit in-depth study by comparing the cytoplasmic and nuclear assets of the oocytes derived from the FEO system, considering that the factors that induce the proper formation of female pronuclei develop and accumulate in the germinal vesicle during oocyte growth [69,121,121,125]. On the other hand, it should be considered that during the transition from PA to EA, only a small number of oocytes are recruited to the final developmental stage by reaching the fully grown size. A process characterized by a structural, functional, and complete biochemical arrangement of the chromatin [126] is essential to support a correct process of resumption of meiosis and subsequent oocyte activation. As previously demonstrated in a study conducted by our group [126], during the PA to EA follicular transition, the GV assumes a nuclear organization similar to that of the fully grown oocytes isolated from preovulatory follicles. In addition, GV showed a progressive organization of large-scale chromatin configuration in a manner in which condensed chromatin surrounds the nucleolus of sheep oocytes isolated from EA follicles. However, differently to the mouse model [127], the surrounding nucleolus configuration did not represent the more advanced configuration toward ovulation since medium and preovulatory antral follicles developed a new pattern of GV organization, where the condensed chromatin appeared localized partly around the nucleolus and partly close to the nuclear envelope (SNE pattern) [126,127,128,129]. Therefore, this incomplete chromatin reorganization could explain the reason why a conspicuous number of oocytes deriving from EA follicles were activated but never overcame the PN stage (see Figure 9B). If the OSE are capable of increasing the rate of maturation via the somatic component of the follicle, the data from the PN stage may indicate the need for the adoption of cultural pre-maturation methods, which might help the germinal component to synchronize its metabolic assets prior to maturation induction.

Overall, the present work demonstrates that EA-derived oocytes can be matured if culture systems are developed to compensate for their low competence. Indeed, for the first time, it was possible to demonstrate that the maintenance of the mural component of the follicle, preserved thanks to the use of the REPROTEN-FEO system and the synergistic dialogue with the OSE cells in coculture, has enhanced the resumption of meiosis and, at the same time, a higher performance of parthenogenetic activation. However, the cytoplasm maturation promoted by the REPROTEN-FEO system did not exceed 30% of FEO-matured oocytes besides the higher activation percentage.

Notably, the failure in activation of FEO-matured oocytes makes them an interesting model to study the signaling pathways activated by the OSE in promoting gamete maturation and, at the same time, to prove the molecular defects responsible for the PN stage block that may limit the transition of low competent derived zygotes towards the early stage of embryo development.

## 4. Materials and Methods

### 4.1. Chemicals

Unless otherwise indicated, all the chemicals used in this study were purchased from Sigma (Sigma Chemical Co., St. Louis, MO, USA).

### 4.2. Ethic Issues

No ethical issues are faced for the present research since all the biological materials were obtained from tissues of feed chain animals discarded by the local slaughterhouse.

### 4.3. Poly (Epsilon-Caprolactone) (PCL) Patterned Electrospun Scaffolds Fabrication

Patterned oriented scaffolds, previously validated for ovarian engineering protocols [77,78], were produced by using a polymeric solution for the electrospinning, prepared by dissolving PCL (average Mn 80,000) in glacial acetic acid (VWR, Darmstadt, Germany), with a concentration of 20% *w*/*v*, stirred overnight and electrospun directly after immersion for 1 h in an ultrasound bath. The process parameters used for the PCL-Patterned scaffold are standardized [77] and briefly reported here: the applied voltage was set at 15 kV, the distance between the tip of the needle and the collector was 11 cm, the flow rate of the solution was 0.4 mL/h, and the needle diameter was 23 G. The used electrospinning device was equipped with a climate chamber, which allowed the setting and control of temperature and relative humidity during the process (EC-CLI, IME Medical Electrospinning, Waalre, The Netherlands).

The electrospun fiber morphology scaffold topology was always tested before use using a scanning electron microscope (SEM) (Auriga Base, Zeiss, Germany).

### 4.4. Biological Samples Recovery

#### 4.4.1. Ovaries Collection

The present research was carried out by collecting Appenninica sheep lamb ovaries from discarded tissues. The ovaries derived from pre-pubertal sheep (about 5 months old) were picked from animals intended for consumption.

The ovaries were collected from the local slaughterhouse and transported to the laboratory in a thermostatic container to control the temperature during the transportation from the slaughterhouse to the laboratory, usually within 1 h. The ovaries were immediately rinsed several times in NaCl 0.9% solution supplemented with Benzoxonium chloride 1 mg/mL (Cat. No. 032186013 Bialcol Med, Vemedia Pharma S.r.l., Parma, Italy). After medulla removal, the ovaries were transferred into a HEPES-buffered TCM199 medium (Cat. No. M7528) and cut into regular-sized cortical fragments (approximately 0.5 × 0.5 × 0.5 cm).

#### 4.4.2. Cell Collection for Coculture System Setup

The IVM and FEO maturation protocol was in the absence or presence of primary cell monolayer coculture. More in detail, both COCs and whole follicles derived from EA structures were incubated on confluent monolayers of cells isolated from the ovary (ovarian surface epithelium-OSE and granulosa cells-GC) or non-ovarian district (tenocytes-TC). More in detail, OSE cells, previously validated by our group as cell monolayer coculture (positive Ctr) [78,80], were isolated using a surgical scalpel from the ovarian cortex of pre-pubertal ovaries previously incubated in 0.25% Trypsin/EDTA 200 mg/L at 38.5 °C for 5 min. Cell suspensions were collected into a petri dish (6 cm) filled with DPBS solution containing 30% Fetal bovine serum (FBS) (Gibco, Darmstadt, Germany) to inactivate Trypsin, centrifuged, and the supernatant discarded.

GCs were isolated from medium-antral follicles walls (mural GC) using tissue forceps and collected into a petri dish (6 cm) filled with DPBS solution. Then, the cell suspension was centrifuged, and the supernatant was discarded.

OSE and GC were seeded for cell expansion in a 10 cm Petri dish until reaching 60–80% of confluence.

TCs were isolated after in vitro incubation of fetal tendon explants obtained by fetuses of 25–35 cm of length at ~2–3 months of pregnancy, derived from slaughtered animals Appenninica breed sheep by using 0.25% Trypsin/EDTA solution when the cells migrated out of tendon pieces and reached 60–80% of confluence, as previously described [130,131,132].

The concentration of vital cells was defined after pellet resuspension and Trypan Blue staining via a hemocytometer chamber. All cellular sources were cultured in a growth medium consisting of alphaMEM (Cat. No. BE02-002F Lonza, Gampel-Bratsch, Switzerland), 20% fetal bovine serum (FBS: Cat. No. 11573397 Gibco), 1% glutamine (Cat. No. BE17-605E/U1 Lonza), antibiotics such as 75 mg/L of penicillin-G and 50 mg/L of streptomycin sulfate (Cat. No. DE17-602E Lonza). After one expansion passage, OSEs, GCs, and TCs were seeded according to the experimental group described in Section 2.1.2, Section 2.2.2 and Section 2.2.4 and used for coculture trials once they reached 50–60% of confluence.

#### 4.4.3. In Vitro Maturation (IVM) of Cumulus Oocytes Complexes (COCs) Isolated from Early Antral (EA) Follicles

The COCs were mechanically isolated from EA follicles (mean diameter: 360 ± 8 μm) using 32 G sterile needles under a stereomicroscope in the flow hood, and the ones presenting continuous and compact layers of cumulus cells were selected to perform IVM protocols.

The COCs were divided into 4-well plates (Nunc, Roskilde, Denmark) and left in a maturation culture medium, adopting a previously validated protocol [80]. Each well of the 4-well plate was filled with 500 μL of maturation medium: alphaMEM, 20% fetal bovine serum (FBS: Cat. No. 11573397 Gibco), 1% glutamine (Cat. No. BE17-605E/U1 Lonza), antibiotics like 75 mg/L penicillin-G, and 50 mg/L streptomycin sulfate.

To assess the inductive maturation role of chorionic gonadotropins (human Chorionic Gonadotropin/hCG and equine Chorionic Gonadotropin/eCG) in IVM of low competent oocytes derived from EA follicles, a dedicated experiment was set up using previously validated concentrations of hormones [78,80]. To this aim, COCs were divided into 4 groups consisting of at least 40 COCs each and treated (1) without hormones (Ctr) or with (2) 5 IU/mL hCG + 5 IU/mL eCG, (3) 5 IU/mL hCG, and (4) 5 IU/mL eCG in three different replicates.

COCs were left in an incubator at 38.5 °C and 5% CO_2_, and the oocyte maturation performances were assessed after 24 h of culture.

A second setup of experiments was designed to assess the influence of cell coculture by dividing the COCs into 4 groups consisting of at least 40 oocytes. Each group was incubated (1) in the absence of cells (negative Ctr) or on sub-confluent monolayers of different cell types: (2) OSE (positive Ctrl; previously validated [78,80]), (3) GC, and (4) TC in 3 experimental replicates. The COCs were left in the maturation medium previously described for 24 h before assessing the nuclear stage.

#### 4.4.4. Maturation of Follicle Enclosed Oocyte (FEO) Derived from EA Follicles

The EA follicles were mechanically isolated from the cortical fragments using 32 G sterile needles under a stereomicroscope in the flow hood and selected based on their morphology and size in order to keep the theca layer intact.

The EA follicles isolated for FEO maturation experiments displayed a mean diameter of 360 ± 14 μm. The EA follicles were accurately analyzed before incubation under an inverted-phase microscope associated with the time-lapse imaging software, NIS-Elements Advanced Research software 4.51.00 (Eclipse Ti Series, Nikon Europe BV, Amsterdam, The Netherlands), to determine its diameter and to exclude any morphological sign of degeneration such as somatic cell or oocyte darkness, loss of compactness of granulosa layer or basal membrane break.

The selected EA follicles were randomly divided into different groups and placed on trans-well culture systems using 96-well plates with U-shaped wells and with the holder filled with PCL-Patterned electrospun scaffolds as previously described [78]. The trans-well systems were filled with 100 μL of maturation medium: alphaMEM (Cat. No. BE02-002F Lonza), 20% fetal bovine serum (FBS: Cat. No. 11573397 Gibco), 1% glutamine (Cat. No. BE17-605E/U1 Lonza), antibiotics such as 75 mg/L of penicillin-G and 50 mg/L of streptomycin sulfate (Cat. No. DE17-602E Lonza).

A preliminary experiment was performed to assess the use of PCL-Patterned electrospun scaffolds for the culture of EA-FEO purpose (overall 30 FEOs). EA follicles were left in an incubator at 38.5 °C and 5% CO_2,_ and the CG effect on oocyte maturation performances was assessed after 24 h of IVM and finally analyzed morphologically.

Firstly, the hormonal trigger of oocyte maturation was optimized for FEO protocol. To this aim, a preliminary dose-effect experiment was set up using groups of at least 40 EA follicles. Each group was exposed to an increasing dose of hCG (0, 5, 25, 50 IU/mL) in three different replicates.

The second step was aimed to test the contribution of chorionic gonadotropins on FEO by exposing groups of at least 30 EA FEO to (1) 5 IU/mL eCG, (2) 25 IU/mL eCG, (3) 50 IU/mL eCG, (4) 5 IU/mL eCG + previously determined dose of hCG, (5) 25 IU/mL eCG + hCG, (6) 50 IU/mL eCG + hCG, (7) hCG alone, (8) Ctr (no hormones). Three different replicates were performed.

Finally, once the hormonal stimulation for triggering maturation has been set up, the EA FEO maturation protocol was performed on different cell cocultures. Four groups consisting of at least 40 EA follicles were incubated over (1) OSE, (2) GC, (3) TC monolayers, or (4) without cells in two different trials. The EA follicles were left in the maturation medium previously described for 24 h in an incubator at 38.5 °C and 5% CO_2_ prior to the treatment for the nuclear-stage assessment.

#### 4.4.5. Comparison between IVM vs. FEO Maturation Protocol of Low Competent Oocytes Derived from EA Follicles

Once the cultural condition for IVM and FEO maturation protocols were set up (chorionic gonadotropin stimulation), comparative experiments were designed to assess the influence of cell coculture on in vitro maturation protocols adopted for COCs (IVM) or for FEO. For each experiment (4 replicates), the groups were built up with at least 30 COCs or 30 FEO, and both cocultured on: (1) OSE, (2) GC, and (3) TC in the presence or absence of optimized hormonal stimulation. Both the maturation protocols were performed for 24 h in an incubator at 38.5 °C and 5% CO_2_ prior to the treatment for the nuclear-stage assessment.

### 4.5. Meiotic and Developmental Competences of Low Competent Oocytes Derived from EA

#### 4.5.1. Oocyte Nuclear Stage Assessment

The meiotic resumption in in vitro matured oocytes under IVM and FEO protocols were denuded of surrounding cumulus cells, permeabilized/fixed in a solution of acetic acid and ethanol (1:3) for at least 12 h, and finally stained with 1% Lacmoid (Cat. No. 274720 Sigma) solubilized in distilled water. Then, the oocytes were mounted on the object slide and analyzed under a Phase Contrast Microscope (AxioVert, Carl Zeiss, Jena, Germany) for the detection of the nuclear stage. Only healthy COCs derived from EA presenting expanded layers of cumulus cells and corresponding oocytes without signs of cytoplasmic degeneration were considered for IVM and FEO experiments.

The oocytes were classified according to the meiotic nuclear stages in Germinal Vesicle (GV), GVBD, Metaphase I (MI), and Metaphase II (MII) according to [79]

#### 4.5.2. Parthenogenetic Activation

To determine the degree of cytoplasmic maturation, parthenotes were produced by activating MII oocytes identified for the extrusion of the first polar body under the stereomicroscope. The parthenogenetic competence assessment was performed on MII oocytes derived from the comparative experiments of optimized IVM vs. FEO maturation protocol (Section 4.4.5) and compared with that of MII oocytes isolated from medium antral follicles (positive Ctr). Parthenogenetic activation was carried out according to a previously validated protocol [79]. After 72 h from activation, both uncleaved and cleaved oocytes were analyzed under an inverted microscope (time-lapse imaging software NIS-Elements (Eclipse Ti Series, Nikon, Japan) to evaluate their developmental stage by counting the number of blastomeres. The Lacmoid staining described in Section 4.5.1 was also performed to confirm the nuclei presence.

### 4.6. Biochemical FEO Analyses

In order to optimize the hCG stimulation in EA-FEO and confirm the specificity of cell co-cultural influence on this maturation protocol, follicle walls were analyzed as described below.

#### 4.6.1. Real-Time qPCR

The total RNA was extracted with a Single-Cell RNA Purification Kit (Norgen Biotek Corp. Cat 51800) following the manufacturer’s instructions. A total of 1 μg of total RNA was retrotranscribed using oligodT primers (Bioline, London, UK) and Tetro Reverse Transcriptase (Bioline, London, UK), following the manufacturer’s instructions. The qPCRs were carried out in triplicate using the SensiFAST SYBR Lo-ROX kit (Bioline London, UK) on a 7500 Fast Real-Time PCR System (Life Technologies, Carlsbad, CA, USA), according to the manufacturer’s instructions. The following PCR conditions were used for all the experiments: 95 °C for 10 min, followed by 40 cycles at 95 °C for 10 s and 60 °C for 30 s. Relative quantification was performed by using the ∆∆Ct method. GAPDH (Glyceraldehyde 3-phosphate dehydrogenase) and YWHAZ (Tyrosine 3-Monooxygenase/Tryptophan 5-Monooxygenase Activation Protein Zeta) were selected amongst the housekeeping genes for gene quantification. The expression profiles were similar with both reference genes. The primer sequences are reported in Table 1.

#### 4.6.2. Quantification of Protein Expression

The cell extracts were obtained from EA follicular walls collected and homogenized in RIPA buffer (R0278, Sigma-Aldrich, St. Louis, MO, USA) supplemented with protease (P2714, Sigma-Aldrich, St. Louis, MO, USA) and phosphatase (P5726, Sigma-Aldrich, St. Louis, MO, USA) inhibitors.

Cell extracts of samples were put on ice for 30 min and centrifuged at 12,000× *g* for 10 min at 4 °C, and then protein concentration was determined by using Quick Start™ Bradford 1x Dye Reagent (Bio-Rad Laboratories, Hercules, CA, USA). Next, 20 μg of total protein extracts from each sample were denatured in Laemmli Sample buffer before SDS-PAGE and used for Immuno-blot analysis.

Anti-bTubulin (T5168, Sigma-Aldrich, St. Louis, MO, USA), anti-pEGFR (sc-12351, Cell Signaling Technology, Danvers, MA, USA), anti-EGFR (2232, Cell Signaling Technology, Danvers, MA, USA), anti-pMEK (9121, Cell Signaling Technology, Danvers, MA, USA), anti-MEK (9122, Cell Signaling Technology, Danvers, MA, USA), anti-pERK (sc-7383, Santa Cruz Biotechnology, Dallas, TX, USA), anti-ERK (sc-154, Santa Cruz Biotechnology, Dallas, TX, USA). HRP-conjugated secondary antibodies were mouse (sc-516102, Santa Cruz Biotechnology, Dallas, TX, USA), rabbit (sc-2357, Santa Cruz Biotechnology, Dallas, TX, USA), and goat (sc-2354, Santa Cruz Biotechnology, Dallas, TX, USA). Incubation with antibodies was performed according to the manufacturer’s instructions. Azure 400 Imager (Azure Biosystems, Dublin, CA, USA) was used to reveal chemiluminescent signals on the membranes. Densitometric analysis for protein quantification was performed with Image J blot analyzer software (ImageJ 1.53 k, NIH, Bethesda, MD, USA).

### 4.7. Statistical Analysis

Three independent experimental replicates were performed. The data are presented as the percentage or mean ± SD. GraphPad Prism 9 (GraphPad Software) was used for the statistical analyses, and values with *p* < 0.05 were considered statistically significant.

Differences in the achievement of oocyte meiotic competence and parthenogenetic development in vitro between different groups were evaluated by ordinary one-way ANOVA followed by the Dunnett test to compare multiple groups. All the other data were analyzed by an unpaired *t*-test.

## Figures and Tables

**Table 1:** Sequences of primers used in real-time qPCR.

Gene	Forward Sequence	Reverse Sequence
*CYP19A1*	5′-TCGTCCTGGTCAACCCTTCTG-3′	5′-CCAGACGAGACCAGAGACCG-3′
*GAPDH*	5′-TCGGAGTGAACGGATTTGGC-3′	5′-CCGTTCTCTGCCTTGACTGT-3′
*YWHAZ*	5′-AGACGGAAGGTGCTGAGAAA-3′	5′-CGTTGGGGATCAAGAACTTT-3′

## Data Availability

Data is contained within the article.
